# The Body across the Lifespan: On the Relation between Interoceptive Sensibility and High-Order Body Representations

**DOI:** 10.3390/brainsci11040493

**Published:** 2021-04-13

**Authors:** Simona Raimo, Antonella Di Vita, Maddalena Boccia, Teresa Iona, Maria Cropano, Mariachiara Gaita, Cecilia Guariglia, Dario Grossi, Liana Palermo

**Affiliations:** 1 Department of Psychology, University of Campania ‘Luigi Vanvitelli’, 81100 Caserta, Italy; maria.cropano@studenti.unicampania.it (M.C.); dario.grossi50@gmail.com (D.G.); 2 Department of Human Neuroscience, ‘Sapienza’ University of Rome, 00185 Rome, Italy; antonella.divita@uniroma1.it; 3 Department of Psychology, Sapienza University of Rome, 00185 Rome, Italy; maddalena.boccia@uniroma1.it (M.B.); cecilia.guariglia@uniroma1.it (C.G.); 4 IRCCS Santa Lucia Foundation, 00179 Rome, Italy; 5 Department of Experimental and Clinical Medicine, University ‘Magna Graecia’ of Catanzaro, 88100 Catanzaro, Italy; iona@unicz.it; 6 Department of Medical and Surgical Sciences, University ‘Magna Graecia’ of Catanzaro, 88100 Catanzaro, Italy; mariachiara733@gmail.com

**Keywords:** body representation, body schema, body structural representation, interoceptive sensibility, life span, childhood, aging

## Abstract

Background: Interoceptive information plays a pivotal role in building higher-order cognitive body representations (BR) that neuropsychological and neuroimaging evidence classifies as action-oriented (i.e., body schema) or non-action-oriented (i.e., visuo-spatial body map). This study aimed to explore the development of BR, considering the association with the interoceptive sensibility throughout the lifespan. Methods: Two hundred thirty-nine healthy participants divided into five age groups (7 to 8 years; 9 to 10 years; 18 to 40 years; 41 to 60 years; over 60 years) completed a self-report measure of interoceptive sensibility (the Self-Awareness Questionnaire; SAQ) and were given tasks assessing the two BR (action-oriented: hand laterality task; non-action-oriented: frontal body evocation task). Results: Both children (7–8 and 9–10 years) and older adults (over 60 years) performed worse than young (18–40 years) and middle-aged adults (41–60 years) in action- and non-action-oriented BR tasks. Moderation analyses showed that the SAQ score significantly moderated the relationship between age and action-oriented BR. Conclusions: The current results are consistent with inverted U-shaped developmental curves for action- and non-action-oriented BR. As an innovative aspect, the ability to mentally represent one’s own body parts in diverse states could be negatively affected by higher interoceptive sensibility levels in childhood and late adulthood.

## 1. Introduction

Representing our own bodies in our minds is a complex process. Actually, it relies on processing and integrating stimuli from many different sources of information (e.g., interoceptive, exteroceptive, and motor information), both inside and outside the body, and has profound implications for processing and localizing sensations to perform appropriate actions and to interact with the environment [1,2,3,4]. Several behavioral and imaging studies have investigated high-order body representations (BR) in patients with brain damage [4,5,6,7,8,9] and healthy individuals (for a review, see [10]), suggesting that they may be segregated into dynamic representations of the body, which are mainly devoted to the planning and monitoring of movements (action-oriented BR), and stable representations of the body, which are not functionally specialized to support actions, but are relevant for perception/recognition, body ownership, and self-consciousness (non-action-oriented BR; [10,11]). Specifically, action-oriented BR roughly corresponds to the body schema, defined as the dynamic representations of the body derived from multiple sensorimotor inputs, which interact with the motor system in the genesis of actions [9]; instead, for example, the so-called visuospatial body map, which is a topographical representation of the body derived from visual information, including body part boundaries and proximity relationships [9], is a kind of non-action-oriented BR.

In addition to being affected independently by brain damage [7], different BR may have different developmental trajectories in childhood [12,13,14,15,16,17] and be differently vulnerable to age-related changes as well [18,19,20,21].

In particular, a study investigating both action- and non-action-oriented BR in the same sample of school-aged children showed that the structural representation of the body reached an adult-like pattern by the age of 9–10 years, whereas the body schema was still not completely matured and would continue to develop during the school-age [16]. Studies using the mental rotation of body parts, which is a task probing the body schema, suggest that an adult-like performance is reached at 12 years since only subtle differences are observed between adolescents and adults [22].

Moreover, a study of Cardinali and colleagues [23], conducted on a large sample of children from 6 to 10 years old (*N* = 84), found that a hand size underestimation was already present in the youngest children and that it was functional for proper interaction with the external environment. Instead, a recent developmental study, investigating action- and non-action-oriented BR in two different experiments with two different samples of participants (*N* = 56 and *N* = 69 respectively), found no difference between participants in the pre-growth spurt (mean age 8.5 years), the growth spurt (mean age 12.8 years) and the post-growth spurt (mean age 39.4 years; age range 15–50 years) periods in adapting reaching movements to the changes in affordance or in estimating the tactile distance between two simultaneously applied tactile stimuli on the arm [24].

On the other hand, after 60 years of age, behavioral studies on healthy participants suggest a decline of both types of BR (action-oriented BR [19,20,25,26] and non-action-oriented BR [18,20,21]). Despite this evidence, whether (and to what extent) different BR can be differently affected by physiological aging is still debated. Indeed, for example, a recent account [21] of the age effect in perceiving the metrics of the upper limb (i.e., a non-action-oriented BR) and peripersonal space (i.e., a space that is functionally linked to the body schema; see [25,27] for an overview on the topic and evidence of distinctions and commonality) suggests an age effect on the non-action-oriented BR, but no age effect on a representation involved in acting on the space immediately surrounding our body.

To summarize, the current studies do not allow us to conclude on a strong effect of age on these BR, as other factors, such as individual differences, the sensitivity of the tasks, and spurious effects can have important roles. Indeed, a limitation of the majority of previous developmental (e.g., [13,15,17,24]) and aging (e.g., [21,25,26]) studies was in the assessment of only one kind of BR (e.g., [13,15,17,26]) or in the evaluation of different BR in different groups of participants (e.g., [21,24]). Additionally, the majority of previous studies used only BR tasks (e.g., [13,14,17,21,22,24]), while control tasks, which allow taking into account possible spurious effects due to other cognitive skills or task difficulty, were not given to participants. Indeed, a potential fallacy in the lifespan studies is erroneously attributing low performance to the age effect. For example, low performance in an action-oriented BR task, such as a mental rotation of body parts, could be due to a more general decline in transforming mental images (see [28]) and not in a specific difficulty in the body schema. One of the strategies to prevent such a risk is to compare performance in a control task similar to the experimental one in terms of setting (stimuli presentation; response modality) and task difficulty (see [16]). To date, no study has explored BR from a lifespan development perspective, with a comprehensive assessment of action- and non-action-oriented BR within the same sample of participants and using both BR and control tasks.

Moreover, little attention has been paid to the possible interplay between BR and interoceptive information, which can play a pivotal role, together with other top-down and bottom-up sources of information, in BR processing [4,29].

Interoception refers to the processing of internal bodily stimuli [30]. It includes different dimensions, such as (see [30]): the interoceptive attention (i.e., the process of observing internal bodily sensations), the interoceptive accuracy or sensitivity (i.e., the process of correctly and precisely monitoring the sensations as assessed by comparisons between subjective and objective indices), and the interoceptive sensibility (i.e., the self-perceived tendency to focus on interoceptive signals, which represents a trait-like feature). Previous studies on the relation between BR and interoception mainly focused on interoceptive accuracy [31,32]. Overall, they showed that a higher awareness of one’s inner body sensations would decrease the plasticity of the body representation [33]. However, little is known about the role played by interoceptive sensibility on BR, and so far, only one study showed that the tendency to focus on one’s own body sensations significantly affects the action-oriented BR processing in older age [20].

Despite being theoretically relevant, a better understanding of BR across the life span and of the role of the interoceptive sensitivity is also necessary to improve our knowledge of BR deviations from typical development and physiological aging in clinical populations in order to provide tailored rehabilitation training programs for specific BR deficits. Additionally, BR deficit following a stroke or cerebral palsy can have a huge impact on motor rehabilitation, since BR interact with the motor system in the genesis of actions (for such an argument, see [7,34]). 

Therefore, this study aimed to explore BR lifespan changes in a large sample of individuals whose ages ranged between school-age and late adulthood; the role played by the processing of interoceptive information throughout the lifespan was assessed as well.

## 2. Materials and Methods

### 2.1. Participants

Two hundred and thirty-nine healthy individuals participated in this study. They were grouped into five age bands based on two main reasons. First, we wanted to be sure that participants could clearly understand instructions and perform the task, so we decided to sample children of an age range centered around 8 years old (for a similar methodology, see [23]). Then, we divided them into two groups: 65 typically developing children from 7 to 8 years old (31 girls and 34 boys; 56 right-handed), and 37 typically developing children from 9 to 10 years old (24 girls and 13 boys; 30 right-handed). This group division was relevant since previous literature showed that children until 8 years of age do not reach optimal multisensory integration of visual and haptic information [35,36], do not reach an adult-like pattern regarding localizing body parts [16], and do not show significant differences in BR compared to children with atypical development [34]. 

Second, we divided the adult sample into three age bands in line with previous studies on age-related cognitive changes in adulthood [37,38,39] and findings on the ability to mentally manipulate body parts. Indeed, previous studies showed that the ability to mentally manipulate body parts is fully operative from late adolescence (17–18 years), and there are only subtle improvements between adolescents (13–17 years) and adults [40,41]. Accordingly, the group of young adults consisted of 50 participants aged from 18 to 40 (25 females and 25 males; 41 right-handed); the group of middle-aged adults consisted of 50 participants aged from 41 to 60 (30 females and 20 males; 42 right-handed); and the group of older adults consisted of 37 participants aged over 60 (26 females and 11 males; 32 right-handed). 

All participants were native Italians from an urban context in the South of Italy. 

In particular, children were recruited from state schools in Calabria (Italy); none of them had repeated any year of school; none of them had a statement of special educational needs, had developmental or learning difficulties, had been diagnosed with a neurological condition, or had ever shown any emotional or behavioral condition based on parental and/or teacher reports. All children showed normal reasoning ability according to the Italian norms of the Colored Progressive Matrices [42,43]. 

Adult participants were recruited by word-of-mouth from “Magna Graecia” University, Catanzaro (Italy), and from the Psychology Department of University of Campania “Vanvitelli”—Caserta (Italy). None of them had current mental health disorders, such as depression and anxiety, according to the Diagnostic and Statistical Manual of Mental Health Disorders, 5th Edition (DSM-5; [44]). All adult participants obtained normal age- and education-adjusted scores on the Mini Mental State Examination (MMSE; [45,46]) and on the Raven’s Coloured Progressive Matrices [42,47], which excluded the presence of general cognitive impairment and any deficit in abstract reasoning. 

All healthy adults and children’s parents signed informed consent, and children’s assent was also received before the investigation. The sample overlapped with those of previous studies of our research group [16,20]. Approval was obtained from the local ethics committee: Calabria Region Ethical Committee, Catanzaro, Italy (protocol number 311, 21 December 2017), and Ethical Committee of the University of Campania “Vanvitelli,” Caserta, Italy (protocol number 695, 10 January 2018) in accordance with the criteria set laid down in the 1964 Declaration of Helsinki.

### 2.2. Behavioral Testing

#### 2.2.1. Assessment of the Interoceptive Sensibility

To assess interoceptive sensibility, participants completed the Self-Awareness Questionnaire (SAQ; [48]). It is a self-report questionnaire specifically developed to assess the interoceptive sensibility without considering other body awareness aspects (i.e., the quality of body sensations, the attention regulation for body sensations, the emotional awareness for physiological signs of emotion, or the tendency to evaluate one’s own body as safe and trustworthy) that takes into account in other questionnaires (e.g., the Multidimensional Assessment of Interoceptive Awareness; [49]). It is composed by 35 items to be rated on a 5-point Likert (0 = never; 1 = sometimes; 2 = often; 3 = very often; 4 = always) measuring how frequently signals from the body are felt by respondents. The total score was the sum of the responses of all 35 items providing a score range of 0 to 140, with higher scores indicating higher levels of interoceptive sensibility and focusing on one’s own bodily sensations. Since SAQ has not been validated for use with children, we re-worded, without changing the meaning, some of the items to facilitate understanding. In particular, the original item 5, “My head feels empty,” was re-worded as follows: “My head feels empty as though without thoughts”; the original item 16, “I feel as if I am on fire,” was re-worded as follows: “I feel so hot as if I was near a flame, or as if I was a fire”; the original item 23, “I feel chilled,” was re-worded as follows: “I feel so cold as if I were an icicle”; the original item 26, “I have a heavy feeling in my chest,” was re-worded as follows: “I have a heavy feeling in my chest, as if something heavy is crushing it”; the original item 27, “I feel my heart thudding,” was re-worded as follows: “I feel a blow to the heart.” Moreover, we performed the internal consistency reliability test (Cronbach’s alpha = 0.87) that was similar to that of the original adult version (Cronbach’s alpha = 0.88). 

#### 2.2.2. Assessment of Body Representations

According to the distinction of BR into action- and non-action-oriented [10], the assessment of BR was performed using a specific computerized battery previously used to assess BR in healthy children and adults [16,20], and in children and adults with brain damage [5,7,34]. The battery included BR tasks to evaluate both action (i.e., body schema) and non-action (i.e., body structural representation) oriented BR, and two corresponding control tasks, similar to those recording BR for features such as presentation and response modalities, but not including body stimuli and then not involving body processing. All healthy adults were evaluated in a quiet experimental room at the university, while children were evaluated in a quiet room at their school. All tasks were performed on a laptop (13.3″ display) equipped with a touch screen monitor, and participants were invited to sit on the chair in front of a desk with the laptop placed upon it. During testing, they were instructed to maintain the same position with both hands placed on their knees before producing the response. No time limit was imposed, but they were solicited to respond immediately after the presentation of stimuli.

##### Assessment of the Action-Oriented BR

Action-oriented BR (i.e., body schema) was assessed using the hand laterality task (adapted and simplified from [50]; see [16]). In this task, participants were asked to make a decision, as rapidly and accurately as possible, of the laterality of a single hand (20 stimuli, 10 left and 10 right stimuli), which could be presented at varying degrees of angles of rotation (0, 45, 90, 270, or 315 degrees) by mentally rotating it. 

The control task, the object laterality task (see [16]), included a mental rotation of a non-body stimulus (i.e., a flower with a leaf positioned at the right or at the left base of the stem). Participants were asked to make a decision, as rapidly and accurately as possible, on the laterality of this non-body stimulus (20 stimuli, 10 left and 10 right stimuli), which could be presented at varying degrees of angles of rotation (0, 45, 90, 270, or 315 degrees) by mentally rotating it. 

In both tasks, accuracy was digitally recorded, and individual accuracy corresponded to the sum of correct responses; thus, individual scores ranged from 0 to 20, with higher scores indicating better performance. Participants executed two practice trials to ensure that they understood the instructions. The task presentation order was counterbalanced across participants, and the presentation order of stimuli was consistent within all tasks.

##### Assessment of Non-Action Oriented BR

Non-action-oriented BR (i.e., body structural representation) was assessed using a computerized version of the “frontal body evocation task” (FBE) of the body representation test ([51]; see [16]). Participants were shown the picture of a human body for 10 s, and subsequently, they were asked to re-locate one at time nine specific body parts (left or right leg, left or right hand, left or right arm, left or right part of the chest, and the neck) dragging them with a finger on a touchscreen where only the head was shown as a reference. Participants were presented with one specific body part at a time, and before presenting a new body part, the computer recorded the position of the located body part. The control task, the Christmas tree task, involved the visuo-spatial processing of non-body-related stimuli (see [16]). Participants were shown the picture of a Christmas tree for 10 s, and subsequently, they were asked to re-locate one at time nine specific parts of the tree (left or right lower branches, left or right middle branches, left or right lower branches with trunks, left or right parts of the jar, and the top), dragging them with a finger on a touchscreen where only the star tree topper was shown as a reference. Participants were presented with one specific Christmas tree part at a time, and before presenting a new Christmas tree part, the computer recorded the position of the located part. 

In both tasks, accuracy was computed as the deviation (in millimeters, mm) from the correct location (a smaller deviation in mm indicated a better performance). The task presentation order was counterbalanced across participants.

#### 2.3. Statistical Analysis

##### 2.3.1. Comparison Analyses among Age Groups

To verify the normality of data distribution for accuracy scores, we used the Kolmogorov–Smirnov test. Due to the non-normal distribution of continuous variables (BR and control tasks and SAQ total scores), non-parametric analyses were performed.

To evaluate the presence of differences in the SAQ total scores among the five age groups, we used the Kruskal–Wallis test.

To evaluate differences in BR tasks performances, considering the role of general cognitive abilities required to perform BR task, we performed rank analyses of covariance (Quade’s test) on the accuracy of performance in the hand laterality task and the FBE, with five age groups (young children: 7 to 8 years old; older children: 9 to 10 years old; young adults: 18 to 40 years old; middle-aged adults: 41 to 60 years old; older adults: over 60 years old) as a between-subject factor, and performance obtained in the object laterality task and the Christmas tree task as covariates. To analyze significant effects, Mann–Whitney *U* tests were performed, and a Bonferroni correction for multiple comparisons was applied.

Further comparison analyses on BR tasks performances were also performed among six age groups, by dividing the group of young adults into two subgroups (the first one with participants aged from 18 to 30 years old; and the second one with participants from 31 to 40 years).

In addition, comparison analyses among the five age groups for response times and differences of performance related to the kinds of stimuli in both the hand laterality tasks (i.e., the right/left hand rotated at 0°, 45°, 90°, 270°, or 315°) and the FBE (i.e., left or right leg, left or right hand, left or right arm, left or right part of the chest, and the neck). 

See Supplementary Materials for results of these further comparison analyses.

##### 2.3.2. Correlation Analyses between BR and Interoceptive Sensibility

Correlation analyses were performed to investigate the association between BR and interoceptive sensibility across the lifespan. First, correlations between SAQ total scores and BR and control tasks performances were assessed over the whole sample using Spearman’s rank correlations. Secondly, correlations between SAQ total scores and the scores of the BR tasks were performed using Spearman’s rank correlation coefficient for each group of age (7 to 8 years old, 9 to 10 years old, 18 to 40 years old, 41 to 60 years old, and over 60 years old). Moreover, to better evaluate a possible association between interoceptive sensibility and the two types of BR in the five age groups—taking into account the other cognitive functions required to perform the BR tasks—for each age group, Spearman’s correlations were also performed between SAQ scores and the unstandardized residuals of the ranks of the body tasks and the ranks of the control tasks (i.e., the unstandardized residuals of the hand laterality task scores for the object laterality task scores, and the unstandardized residuals of the FBE scores for the Christmas tree task scores).

##### 2.3.3. Moderation Analysis

Finally, to assess the moderating role of interoceptive sensibility in the relation between age and BR, moderation analyses were conducted using the bootstrapping technique. The bootstrapping moderation analysis was performed using the PROCESS macro for SPSS [52], a piece of software used for moderation, mediation, and conditional process analyses that utilizes a regression-based path analytic framework or ordinary least squares to estimate moderation models [52]. Age was inputted as the independent variable, BR task scores (hand laterality task and FBE scores) were inputted as the outcome variables, and SAQ scores were inputted as the moderator variable. Significant moderation effects were followed by models controlling for cognitive abilities request to perform body tasks (object laterality task and Christmas tree task scores) to assess the extent to which the interoceptive sensibility’s influence was independent of these covariates. All analyses were performed using SPSS version 23.0 (SPSS, Inc. Chicago, IL, USA), and the significance level was set at alpha <0.05.

## 3. Results

### 3.1. Comparison Analyses among Age Groups

**Figure 1:**
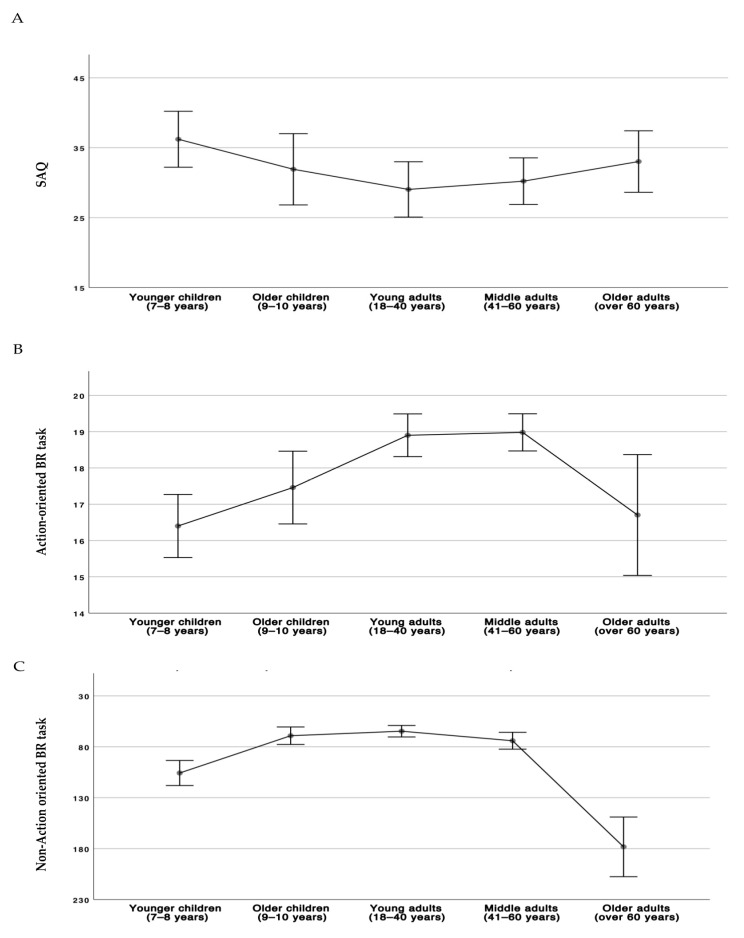
Means with 95% confidence interval error bars in the five age groups for the experimental measures. SAQ score (**A**), action-oriented BR task score, represented by the sum of correct responses (**B**), and non-action-oriented BR task score, represented by the sum of millimeters of deviation from the correct location of each body part (**C**). In panel C the *Y*-axis is inverted for easier comparison to panel B. SAQ, Self-Awareness Questionnaire; BR, body representation.

Means with error bars for SAQ total score and for performance of the BR tasks for the five age groups are shown in Figure 1.

The Kruskal–Wallis analyses showed a non-significant main effect of the age group on the SAQ total score (χ^2^ = 8.33, *p* = 0.080). Concerning the hand laterality task (action-oriented BR), the rank analysis of covariance (Quade’s test) showed a significant effect of group (F_(4235)_ = 6.33, *p* < 0.0001). The significant effect of the age group was further analyzed with Mann–Whitney U tests that showed that the two groups of children and the group of participants aged over 60 performed similarly (children aged 7–8 years vs. children aged 9–10 years: Mann–Whitney U = 988, *p* = 0.129; children aged 7–8 years vs. participants aged over 60 years: Mann–Whitney U = 999, *p* = 0.150; children aged 9–10 years vs. participants aged over 60 years: Mann–Whitney U = 677, *p* = 0.938), but worse than the group of participants aged 18–40 years (children aged 7–8 years: Mann–Whitney U = 864, *p* < 0.0001; children aged 9–10 years: Mann–Whitney U = 646, *p* = 0.010; participants aged over 60 years: Mann–Whitney U = 679, *p* = 0.022) and the group of participants aged 41–60 years (children aged 7–8 years: Mann–Whitney U = 847, *p* < 0.0001; children aged 9–10 years: Mann–Whitney U = 639, *p* = 0.009; participants aged over 60 years: Mann–Whitney U = 679, *p* = 0.023). No difference was found between the group of participants aged 18–40 years and the group of participants aged 41–60 years (Mann–Whitney U = 1246, *p* = 0.978). Concerning the FBE (non-action-oriented BR), the rank analysis of covariance (Quade’s test) showed a significant effect of age group (F _(4235)_ = 40.67, *p* < 0.0001). The significant effect of the age group was further analyzed with Mann–Whitney U tests that showed that the group of participants aged over 60 performed worse than the group of children aged 7–8 years (Mann–Whitney U = 432, *p* < 0.0001); moreover, both groups performed worse than the group of participants aged 18–40 years (children aged 7–8 years: Mann–Whitney U = 629, *p* < 0.0001; participants aged over 60 years: Mann–Whitney U = 64, *p* < 0.0001), the group of participants aged 41–60 years (children aged 7–8 years: Mann–Whitney U = 848, *p* < 0.0001; participants aged over 60 years: Mann–Whitney U = 119, *p* < 0.0001), and the group of children aged 9–10 years (children aged 7–8 years: Mann–Whitney U = 524, *p* < 0.0001; participants aged over 60 years: Mann–Whitney U = 69, *p* < 0.0001). No difference was found between the group of children aged 9–10 years and the group of participants aged 18–40, nor between the former and the 41–60 age group (children aged 9–10 years vs. participants aged 18–40 years: Mann–Whitney U = 873, *p* = 0.655; children aged 9–10 years vs. participants aged 41–60 years: Mann–Whitney U = 831, *p* = 0.420; participants aged 18–40 years vs. participants aged 41–60 years: Mann–Whitney U = 1040, *p* = 0.148).

### 3.2. Correlation Analyses between BR and Interoceptive Sensibility

The correlation analysis performed to assess the associations among SAQ total score, BR, and control tasks performances for the whole sample showed no significant correlations between SAQ total score and the object laterality task score (r_rho_ = 0.010, *p* = 0.873). Instead, significant correlations were found between SAQ total score and the hand laterality task score (r_rho_ = −0.149, *p* = 0.021), the FBE (r_rho_ = 0.193, *p* = 0.003), and its control task, the Christmas tree task (r_rho_ = 0.177, *p* = 0.007). The correlation analyses performed separately on the five age groups showed no significant correlations between the SAQ total scores and the BR task scores, as well as between the two BR task scores, in the three groups of participants aged 7–8 years, 18–40 years, and 41–60 years. Instead, significant correlations were found between the SAQ total scores and the FBE score, and the hand laterality task score in the groups of participants aged 9–10 years and over 60 years (see Table 1).

Moreover, a correlation between BR tasks (i.e., between the hand laterality task and the FBE) was found in participants aged over 60 years and in participants aged 9–10 years (see Table 1). In brief, we found that the higher the interoceptive sensibility, the worse the participants performed on the tasks assessing the action- and non-action-oriented BR, and this effect was specific for the participants aged over 60 years and aged 9–10 years. In these two groups, BR seemed to be related; that is, better performance in the hand laterality task was associated with better performance in the FBE. Correlation analyses between the SAQ total score and the unstandardized residuals of the BR tasks on the respective control tasks confirmed that the interoceptive sensibility was significantly correlated with the BR processing (hand laterality task score: r_rho_ = −0.65, *p* < 0.0001; FBE, r_rho_ = 0.57, *p* = 0.001) in participants aged over 60 years; moreover, significant correlations were found between the SAQ total scores and the unstandardized residuals of the hand laterality task scores on the object laterality task scores in the group of children aged 9–10 years (hand laterality task score: r_rho_ = −0.44, *p* = 0.006). In contrast, no significant correlations were found between the SAQ total scores and the unstandardized residuals of the BR tasks on the respective control tasks in the three groups of participants aged 7–8 years, 18–40 years, and 41–60 years (see Table 2).

### 3.3. Moderation Role of Interoceptive Sensibility in the Relation between Age and BR

**Figure 2:**
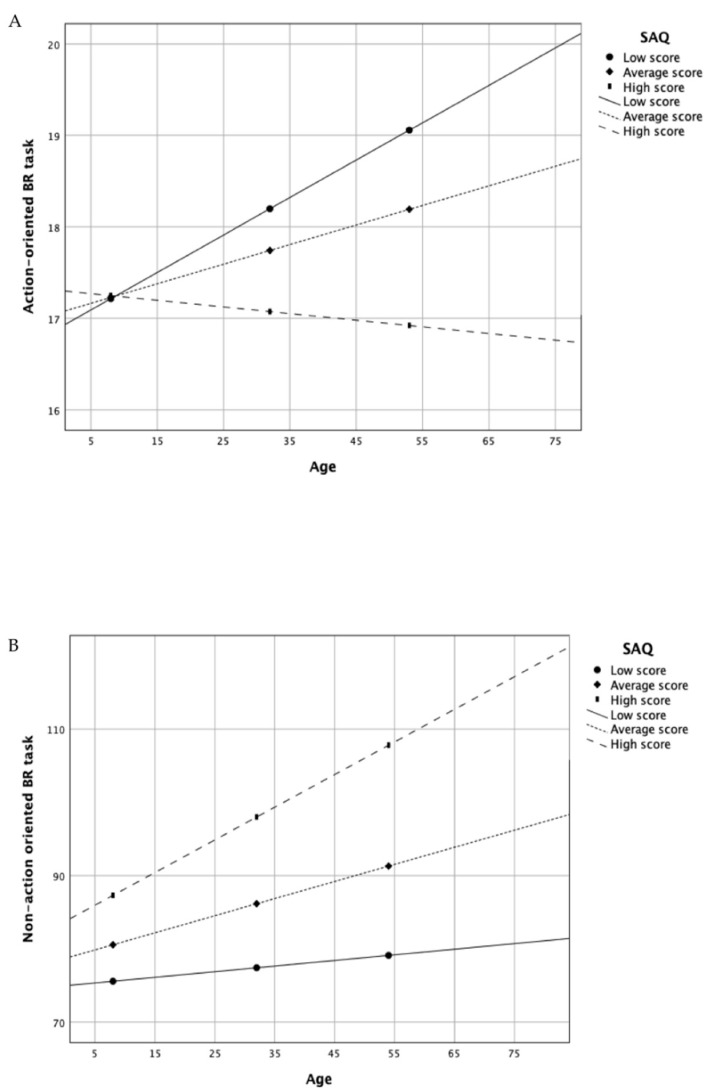
Moderating effects of the interoceptive sensibility on the associations between age and the action-oriented BR task (**A**) and the non-action-oriented BR task (**B**). SAQ, Self-Awareness Questionnaire; BR, body representation.

For the action-oriented BR (hand laterality task), using model 1 in the PROCESS macro for SPSS, the analysis indicated the overall model was significant (*R*^2^ = 0.05, F_(3, 235)_ = 4.44, *p* = 0.004) with a significant interaction between age and SAQ scores (*b* = −0.001, *t* = −2.50, *p* = 0.013). After controlling for cognitive abilities request to perform this BR task (i.e., the object laterality task scores), the overall model remained significant (*R*^2^ = 0.18, F_(4, 234)_ = 12.76, *p* < 0.0001), as did the interaction between the age and SAQ scores (*b* = −0.001, *t* = −2.16, *p* = 0.031). For the non-action-oriented BR, the analysis indicated the overall model was significant (*R*^2^ = 0.07, F_(3, 235)_ = 5.81, *p* = 0.0008), but the interaction between age and SAQ scores was not significant (*b* = 0.012, *t* = 1.50, *p* = 0.133). After controlling for cognitive abilities needed to perform this BR task (i.e., the Christmas tree task scores), the overall model remained significant (*R*^2^ = 0.38, F_(4, 234)_ = 34.84, *p* < 0.0001), but the interaction between the age and SAQ scores was not significant (*b* = −0.001, *t* = −0.07, *p* = 0.941). Accordingly, interoceptive sensibility appeared to significantly moderate only the relationship between age and body schema (see Figure 2).

## 4. Discussion

This study explored the lifespan changes of action and non-action BR in a large sample of healthy individuals from school age to late adulthood, taking into account cognitive functioning and providing a better understanding of the role played by interoceptive sensibility. The most interesting result of this study is represented by the inverted U-shaped age function for BR, with the highest scores being achieved for 18–60, as well as documented for other basic cognitive processes [53,54]. Indeed, our results showed that both children and older adults performed worse than young and middle-aged adults in action-(i.e., body schema) and non-action-oriented (i.e., body structural description) BR tasks. Additionally, we found that non-action-oriented BR reaches its full development earlier than action-oriented BR, since 9–10-year-old children performed similarly to young and middle-aged adults in the body structural representation task, but significantly lower in the body schema task. Therefore, following previous studies on samples of children [13,16,55] and older adults [19,20,21,25], the current findings add valuable and empirical support for the idea that BR rises and falls across the life span. Importantly, these results also clarify whether an age-related effect on BR processing is simply the consequence of an age-related improvement and decline in other cognitive skills (e.g., visuo-spatial processing, mental imagery, and executive functioning [28,53,54]), which in complex tasks, such as BR tasks, can accumulate with significant consequences for performance, or instead it is due to an effect on specific and discrete BR processes with their neuroanatomical substrates. Indeed, using control tasks designed to probe different cognitive skills necessary to perform BR tasks, we have proved that the age effect on BR processing is specific and not ascribable to the overall improvement and decline in other cognitive skills. Results from the correlation analyses provide additional insights into the age-related differences in BR: indeed, we found that action- and non-action-oriented BR were associated with each other and also with interoceptive sensibility in age groups whose BR were not fully developed (i.e., childhood) or had been modified as a consequence of age (i.e., late adulthood). Actually, in 9–10-year-old children and older adults (over 60 years old) groups, performances in task tapping the body schema and body structural representation significantly correlated among them and negatively with the SAQ total score. In agreement with previous behavioral and neuroimaging studies [8,9,10,56], this finding suggests that action- and non-action-oriented BR would be two independent dimensions of BR construct during young and middle adulthood. Instead, this independence can be less marked during development and aging when the abilities to mentally rotate as well as to localize and place correctly body parts are associated among them and negatively affected by the self-perceived tendency to focus on interoceptive signals. Those last conclusions were further confirmed by the results of the moderation analysis, showing that interoceptive sensibility significantly moderated the relation between age and the online sensorimotor and action-oriented representation of the body (i.e., body schema). The connection between interoceptive sensibility and action-oriented BR with developmental and advancing age could be interpreted in light of previous research on body ownership that showed that higher awareness of one’s inner body sensations might affect the plasticity of the BR and make it more difficult to feel ownership for artificial body parts that do not pertain to the physical configuration of the actual body (i.e., the rubber hand Illusion; [32,57]). A similar effect has also been described for the peripersonal space that, as reported in the Introduction, is a concept which, to some extent, overlaps with that of body schema [27,58]. Indeed, a recent study has found that in individuals with higher interoceptive accuracy, the peripersonal space boundaries were narrower and closer to the body [59]. We can hypothesize that the interoceptive processing role in shaping the body schema is even more relevant during childhood and physiological aging. However, when we look at the scientific literature that has considered the processing of a specific kind of interoceptive information, that is, the affective touch, in shaping BR/peripersonal space in healthy adults (for an overview, see [58]), the results are mixed. This suggests that the focus of future studies should be on the possible different weights of various kinds of interoceptive information in shaping different BR across the lifespan. Indeed, interoceptive sensibility questionnaires, such as the SAQ, aim to capture the interoceptive sensibility construct more globally, but the interoceptive processing includes a range of feelings we perceive from our bodies, such as muscular and visceral sensations, hunger, thirst, temperature, pain, itching, and sensual touch [60]. The interoceptive signals that arise within the cardiovascular system, for example, could be more critical for the BR than the interoceptive signals that arise within other systems (e.g., respiratory, gastrointestinal, and urogenital systems) because of the connections between the heart and the brain (i.e., the two most important organs of the body; [33]). 

Overall, our results showed that the action- and non-action-oriented BR have inverted U-shaped developmental curves during one’s lifespan, whose extremities the interoceptive sensibility should play a significant role in affecting BR processing. Additionally, the degree of interaction between different BR could be age-dependent. These findings add new and important support to the co-construction model of Pitron et al. [61], which underlies that although the BR for action and for perception (i.e., body schema and the body image) are functionally distinct, their construction is partly based on their interactions, and that the action-oriented BR, based on multisensory signals and motor expertise, is more affected by the correct processing of interoceptive signals compared to non-action-oriented BR, which is mainly based on visual information [9,62]. 

Despite these new and interesting results, several caveats should be mentioned. First, we examined only some age groups. Our participants were school-age children or adults, making the generalization of our results difficult for individuals of different ages (e.g., preschoolers and adolescents). Additionally, to increase the feasibility of the research program in a large sample of individuals, we used only one experimental task to assess action and non-action BR, and we focused only on the interoceptive sensibility. Thus, starting from our findings, future studies should examine broad age ranges that cover the entire lifespan and assess more extensively BR and the role of different kinds of interoceptive information and of different components of interoceptive processing (i.e., interoceptive attention, interoceptive accuracy, and interoceptive sensibility). In turn, this knowledge will provide deeper insights into the theoretical understanding of BR and will be valuable to improve the assessment and rehabilitation of BR deficits in clinical settings.

## 5. Conclusions

This study has furthered our understanding of BR in two main ways. First, by providing an in-depth analysis of BR across the life span, we have suggested that action- and non-action-oriented BR follow an inverted U-shaped developmental curve, also when the role of other cognitive skills is considered. Secondly, our study has highlighted the importance of taking into account interoceptive sensibility levels that negatively affect, in childhood and late adulthood, the ability to mentally represent one’s own body parts in diverse states from the actual one.

These findings improve the theoretical understanding of BR across the life span and provide support to the co-construction model of BR [61]. Besides, this knowledge also has implications for a better comprehension of BR deviations from typical development and physiological aging in clinical populations in order to develop specific rehabilitation training for BR deficits.

## Figures and Tables

**Table 1:** Correlations between SAQ total score and scores of the action- and non-action-oriented BR tasks for each age group.

**Children Group Aged 7–8 Years**			
		Action-oriented BR task	Non-action-oriented BR task
SAQ	r_rho_ *p*	0.21 0.096	−0.02 0.852
Action-oriented BR task	r_rho_ *p*	-	−0.07 0.581
Non-action-oriented BR task	r_rho_ *p*	-	-
**Children Group Aged 9–10 years**			
		Action-oriented BR task	Non-action-oriented BR task
SAQ	r_rho_ *p*	−0.40 * 0.015	0.36 * 0.028
Action-oriented BR task	r_rho_ *p*	-	−0.36 * 0.027
Non-action-oriented BR task	r_rho_ *p*	-	-
**Healthy Participants Group Aged 18–40**			
		Action-oriented BR task	Non-action-oriented BR task
SAQ	r_rho_ *p*	0.11 0.456	0.02 0.897
Action-oriented BR task	r_rho_ *p*	-	−0.25 0.080
Non-action-oriented BR task	r_rho_ *p*	-	-
**Healthy Participants Group Aged 41–60**			
		Action-oriented BR task	Non-action-oriented BR task
SAQ	r_rho_ *p*	−0.01 0.924	0.10 0.487
Action-oriented BR task	r_rho_ *p*	-	−0.13 0.362
Non-action-oriented BR task	r_rho_ *p*	-	-
**Healthy Participants Group Aged over 60**			
		Action-oriented BR task	Non-action-oriented BR task
SAQ	r_rho_ *p*	−0.68 ** <0.0001	0.67 ** <0.0001
Action-oriented BR task	r_rho_ *p*	-	−0.72 ** <0.0001
Non-action-oriented BR task	r_rho_ *p*	-	-

SAQ, Self-Awareness Questionnaire; BR; body representation. * *p* < 0.05, ** *p* < 0.001.

**Table 2:** Correlations between SAQ total score and the unstandardized residuals of the BR tasks on the respective control tasks.

**Children Group Aged 7–8 Years**			
		Action-oriented BR task	Non-action-oriented BR task
SAQ	r_rho_ *p*	0.21 0.094	0.07 0.602
Action-oriented BR task	r_rho_ *p*	-	0.11 0.412
Non-action-oriented BR task	r_rho_ *p*	-	-
**Children Group Aged 9–10 years**			
		Action-oriented BR task	Non-action-oriented BR task
SAQ	r_rho_ *p*	−0.44 * 0.006	0.05 0.733
Action-oriented BR task	r_rho_ *p*	-	−.23 0.164
Non-action-oriented BR task	r_rho_ *p*	-	-
**Healthy Participants Group Aged 18–40 years**			
		Action-oriented BR task	Non-action-oriented BR task
SAQ	r_rho_ *p*	−0.07 0.628	−0.02 0.913
Action-oriented BR task	r_rho_ *p*	-	−0.07 0.644
Non-action-oriented BR task	r_rho_ *p*	-	-
**Healthy Participants Group Aged 41–60 years**			
		Action-oriented BR task	Non-action-oriented BR task
SAQ	r_rho_ *p*	−0.07 0.624	0.11 0.431
Action-oriented BR task	r_rho_ *p*	-	−0.03 0.819
Non-action-oriented BR task	r_rho_ *p*	-	-
**Healthy Participants Group over 60 years**			
		Action-oriented BR task	Non-action-oriented BR task
SAQ	r_rho_*p*	−0.65 ** <0.0001	0.57 * 0.001
Action-oriented BR task	r_rho_ *p*	-	−0.63 ** <0.0001
Non-action-oriented BR task	r_rho_ *p*	-	-

SAQ, Self-Awareness Questionnaire; BR, body representation. * *p* < 0.05 ** *p* < 0.001.

## Data Availability

Data are available on request.

## Supplementary Materials

The following are available online at https://www.mdpi.com/article/10.3390/brainsci11040493/s1.
