# Supplementary material for: The Body across the Lifespan: On the Relation between Interoceptive Sensibility and High-Order Body Representations
Source: Brain Sci. 2021 Apr 13;11(4):493. doi: 10.3390/brainsci11040493 (PMC8070580; doi:10.3390/brainsci11040493)
Supplement: Supplementary file 1 [file brainsci-11-00493-s001.pdf]

brain sciences

MDPI

Supplementary Materials

Comparison analyses among six age groups

To better investigate BR in young adult participants (aged from 18 to 40 years old), we also performed rank analyses of covariance (Quade's test) on performance in the Hand Laterality Task and in the FBE, with six age groups (young children: 7 to 8 years old; older children: 9 to 10 years old, two groups of young adults: the first one from 18 to 30 years old, and the second one from 31 to 40 years old; middle adults: 41 to 60 years old; older adults: over 60 years old) as the between-subjects factor, and the performance at the Object Laterality Task and at the Christmas Tree Task as covariates. To analyze significant effects, Mann-Whitney U tests were performed.

Concerning the Hand Laterality Task (action-oriented BR), the rank analysis of covariance (Quade's test) showed a significant effect of Group ($F(4,235) = 5.05$, $p < 0.0001$), with the group of young adults aged from 18 to 30 years, and the group of young adults aged from 31 to 40 years showing similar performances to each other(HLT: participants aged from 18 to 30 years=19±1.61; participants aged from 31to 40 years=18.88±2.17; Mann-Whitney U=166, p=0.964), and to the group of middle adults (participants aged from 41 to 60 years; 18-30 years old vs 41-60 years old: Mann-Whitney U=197, p=0.939; 31-40 years old vs 41-60 years old: Mann-Whitney U=1049, p=0.996). Moreover, both groups of younger adults showed significant better performances compared to younger children (participants aged from 7 to 8 years; 18-30 years old vs 7-8 years old: Mann-Whitney U=137, p=0.027; 31-40 years old vs 7-8 years old: Mann-Whitney U=727, p<0.0001), compared to older children (participants aged from 9 to 10 years; 18-30 years old vs 9-10 years old: Mann-Whitney U=82, p=0.029; 31-40 years old vs 9-10 years old: Mann-Whitney U=543, p=0.014), and compared to older adults (participants aged over 60 years; 18-30 years old vs over 60 years old: Mann-Whitney U=63, p=0.023; 31-40 years old vs over 60 years: Mann-Whitney U=571, p=0.026).

Concerning the Frontal Body Evocation Task (non-action oriented BR), the rank analysis of covariance (Quade's test) showed a significant effect of Group ($F(4,235) = 32.45$, $p < 0.0001$), with the group of young adults aged from 18 to 30 years old and the group of young adults aged from 31 to 40 years old showing similar performances to each other (FBE: participants aged from 18 to 30 years = 57.91± 20.66; participants aged from 31 to 40 years = 65.99±19.72; Mann-Whitney U=136, p=0.397), and to the group of older children (participants aged from 9 to 10 years; 18-30 years old vs 9-10 years old: Mann-Whitney U=118, p=0.373; 31-40 years old vs 9-10 years old: Mann-Whitney U=755, p= 0.829), and middle adults (participants aged from 41 to 60 years; 18-30 years old vs 41-60 years old: Mann-Whitney U=132, p=0.125; 31-40 years old vs 41-60 years old: Mann-Whitney U=908, p=0.266). Significant better performances were obtained when they were compared to younger children (participants aged from 7 to 8 years; 18-30 years old vs 7-8 years old: Mann-Whitney U=77, p=0.001; 31-40 years old vs 7-8 years old: Mann-Whitney U=552, p<0.0001), and older adults (participants aged over 60 years; 18-30 years old vs over 60 years old: Mann-Whitney U=6, p<0.0001; 31-40 years old vs over 60 years old: Mann-Whitney U=58, p<0.0001).

Comparison analyses among age groups on response times

To evaluate differences in the speed of performance in the BR tasks, we performed rank analyses of covariance (Quade's test) on the average speed of performance in the Hand Laterality Task and in the FBE, with five age groups (young children: 7 to 8 years old; older children: 9 to 10 years old, young adults: 18 to 40 years old; middle adults: 41 to 60 years old; older adults: over 60 years old) as the between-subjects factor, and the average speed of performance at the Object Laterality Task and at the Christmas Tree Task as covariates. To analyze significant effects, Mann-Whitney U tests were performed.

Concerning the Hand Laterality Task, the rank analysis of covariance (Quade's test) showed a significant effect of the mean response time ($F(4,235)=22.76$, $p<0.0001$).
The significant main effect of the age group was further analyzed with Mann-Whitney U tests that showed that the group of younger children and the group of participants aged over 60 performed similarly (7-8 years old vs over 60 years old: $U=1105$, $p=0.431$), and showed longer mean response times compared to the groups of young and middle adults (7-8 years old vs 18-40 years old: $U=130$, $p<0.0001$; 7-8 years old vs 41-60 years old: $U=650$, $p<0.0001$; over 60 years old vs 18-40 years old: $U=197$, $p<0.0001$; over 60 years old vs 41-60 years old: $U=421$, $p<0.0001$). Moreover, the group of older children showed longer mean response times compared to the group of younger and middle adults (9-10 years old vs 18-40 years old: $U=74$, $p<0.0001$; 9-10 years old vs 41-60 years old: $U=370$, $p<0.0001$). Finally, the group of middle adults showed a longer mean response time compared to the group of younger adults (18-40 years old vs 41-60 years old: $U=887$, $p=0.012$).
Concerning the Frontal Body Evocation task, the rank analysis of covariance (Quade's test) showed a significant effect of the mean response time for the localization of the body parts ($F(4,235)=24.33$, $p<0.0001$). The significant main effect of the age group was further analyzed with Mann-Whitney U tests that showed that the group of younger children had a longer mean response time compared to the group of older children, young and middle adults (7-8 years old vs 9-10 years old: $U=888$, $p=0.037$; 7-8 years old vs 18-40 years old: $U=932$, $p<0.0001$; 7-8 years old vs 41-60 years old: $U=991$, $p=0.001$). Moreover, the group of older adults had a longer mean response time compared to all the other groups for the localization of body parts ($U\leq346$, $p\leq0.0001$). No significant difference in the mean response time was found among groups of older children, young adults, and middle adults for localization of body parts ($U\geq769$, $p\geq0.180$).

Comparison analyses among age groups on performance related to the kind of stimuli

To provide more details on differences of performance among groups related to the kind of stimuli, we performed further analysis on Hand Laterality Task performances, calculating the number of errors made from each age group at each rotation angle considering left and right hand separately (Right Hand rotated at 0°, 45°, 90°, 270°, 315°; Left Hand rotated at 0°, 45°, 90°, 270°, 315°). We found that the five age groups significantly differed in the number of errors in stimuli laterally rotated with respect to the body axis (Right Hand 45°, $\chi^2=20.42$, $p<0.0001$; Right Hand 90°, $\chi^2=20.66$, $p<0.0001$; Left Hand 270°, $\chi^2=39.07$, $p<0.0001$; Left Hand 315°, $\chi^2=19.57$, $p=0.001$), whereas no significant differences were showed in other stimuli ($\chi^2\geq9.31$, $p\geq0.056$). In particular, the groups of young children (7-8 years old) showed a significantly greater number of errors for the lateral rotations in either the right- or left-hand condition compared to the groups of younger adults (18-40 years old, and 41-60 years old) (Right Hand 45°, $U\leq1276$, $p\leq0.010$; Right Hand 90°, $U\leq1211$, $p\leq0.002$; Left Hand 270°, $U\leq993$, $p\leq0.0001$; Left Hand 315°, $U\leq1266$, $p\leq0.004$). The groups of older children (9-10 years old) and the group of older adults showed a higher number of errors compared to the group of young adults (18-40 years old) for the lateral rotation at 45° in the right condition ($U\leq684$, $p\leq0.0001$), and for the lateral rotation at 90° in the right condition ($U\leq752$, $p\leq0.013$) and 270° ($U\leq760$, $p\leq0.010$) and 317° ($U\leq741$, $p\leq0.003$) in left-hand condition compared to the group of middle adults (41-60 years old). Percentages of errors made by each age group at each rotation angle, considering left and right hand separately, are shown in Supplementary Figure 1.
The results suggest that during development and in aging, there would be a specific difficulty with simulating movements away from the body.
To provide more details on differences of performance related to the kind of stimuli of the Frontal Body Evocation task (i.e., the different body parts), we performed a further analysis comparing the five age groups on the accuracy of answer (i.e., mm of deviation from the correct location) on the different body parts (left or right leg, left or right hand, left or right arm, left or right part of the chest, and the neck). We found that the five age groups significantly differed in the correct positioning of all different body's parts (right leg, $\chi^2=16.68$, $p=0.002$; left leg, $\chi^2=16.75$, $p=0.002$; right hand,

χ^2=48.55, p<0.0001; left hand, χ^2=62.81, p<0.0001; right arm, χ^2=49.19, p<0.0001; left arm, χ^2=61.06, p<0.0001; right chest, χ^2=61.86, p<0.0001; left chest, χ^2=66.74, p<0.0001; neck, χ^2=47.53, p<0.0001). In particular, the group of young children (7-8 years old) performed significantly worse in the localization of each body part as compared to the group of older children (9-10 years old; U≤885, p≤0.035) and young adults (18-40 years old; U≤1152, p≤0.011), but for the left chest (7-8 years old vs 9-10 years old: U=943, p=0.090; 7-8 years old vs 18-40 years old: U=1293, p=0.080). The group of young children (7-8 years old) performed significantly worse in the localization of each body part as compared to the middle adults (41-60 years old; U≤1214, p≤0.028), but for the left chest (7-8 years old vs 41-60 years old: U=1512, p=0.615), the right chest (7-8 years old vs 18-40 years old: U=1252, p=0.051; 7-8 years old vs 41-60 years old: U=1412, p=0.284), and the right leg (7-8 years old vs 41-60 years old: U=1515, p=0.629). Moreover, the group of young children showed a better performance in the localization of the neck compared to the group of young adults (U=708, p<0.0001) and to the group of middle adults (U=776, p<0.0001).
The group of older adults (over 60 years old) failed to correctly localize each body part as compared to all the other age groups (U≤705, p≤0.008), but for the left leg (7-8 years old vs over 60 years old: U=926, p=0.324; 9-10 years old vs over 60 years old: U=564, p=0.584; 18-40 years old vs over 60 years old: U=623, p=0.060; 41-60 years old vs over 60 years old: U=754, p=0.509), the right leg (7-8 years old vs over 60 years old: U=910, p=0.266; 41-60 years old vs over 60 years old: U=679, p=0.174) and the neck (7-8 years old vs over 60 years old: U=961, p=0.472; 9-10 years old vs over 60 years old: U=540, p=0.407).
No difference was found between the groups of older children, young adults, and middle adults in the localization of the different body parts (U≥796, p≥0.268), but for the neck, where the adult groups showed lower performance than the group of older children (9-10 years old vs 18-40 years old: U=377, p<0.0001; 9-10 years old vs 41-60 years old: U=422, p<0.0001); and the right leg, where the group of middle adults showed lower performance than the group of younger adults (U=950, p=0.039).
Means for performance related to the kind of stimulus of the Frontal Body Evocation task in the five age groups are shown in Supplementary Figure 2.
Thus, overall, from childhood to young adulthood, the improvement in the structural representation of the body is mainly evident for the limbs, while the performance on the chest is similar for children and young adults. On the other hand, physiological aging mainly affects the structural representation of the upper parts of the body.

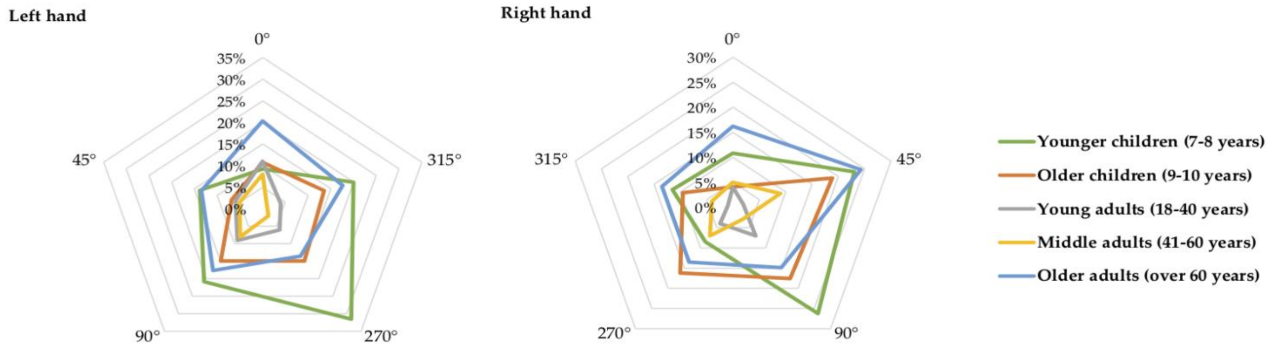

Supplementary Figure 1. Percentage of errors in the Hand Laterality Task made by each age group at each rotation angle and considering left and right hand separately.

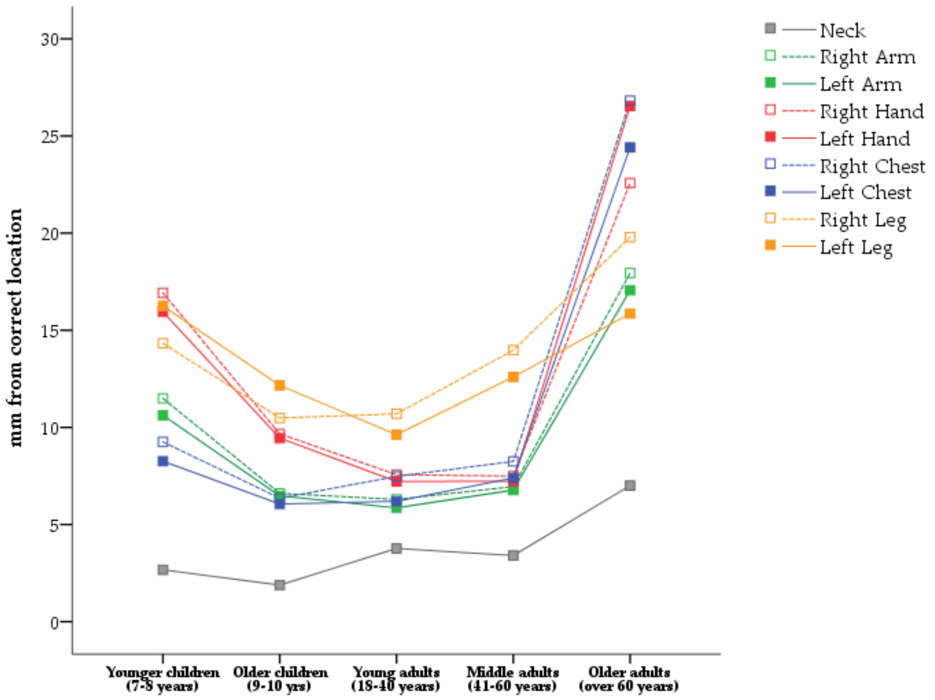

Supplementary Figure 2. Means of performance in the Frontal Body Evocation task for each body part (i.e., average millimeters of deviation from the correct location) in the five age groups.
